# Critical evaluation of the potential of ICP-MS-based systems in toxicological studies of metallic nanoparticles

**DOI:** 10.1007/s00216-024-05181-4

**Published:** 2024-02-08

**Authors:** Sergio Fernández-Trujillo, María Jiménez-Moreno, Nuria Rodríguez-Fariñas, Rosa Carmen Rodríguez Martín-Doimeadios

**Affiliations:** Department of Analytical Chemistry and Food Technology, Faculty of Environmental Sciences and Biochemistry, University of Castilla-La Mancha, Avenida Carlos III s/n, 45071 Toledo, Spain

**Keywords:** Inductively coupled plasma mass spectrometry, Metallic nanoparticles, Toxicological studies, Laser ablation, Single particle/cell, Hyphenated techniques

## Abstract

The extensive application of metallic nanoparticles (NPs) in several fields has significantly impacted our daily lives. Nonetheless, uncertainties persist regarding the toxicity and potential risks associated with the vast number of NPs entering the environment and human bodies, so the performance of toxicological studies are highly demanded. While traditional assays focus primarily on the effects, the comprehension of the underlying processes requires innovative analytical approaches that can detect, characterize, and quantify NPs in complex biological matrices. Among the available alternatives to achieve this information, mass spectrometry, and more concretely, inductively coupled plasma mass spectrometry (ICP-MS), has emerged as an appealing option. This work critically reviews the valuable contribution of ICP-MS-based techniques to investigate NP toxicity and their transformations during in vitro and in vivo toxicological assays. Various ICP-MS modalities, such as total elemental analysis, single particle or single-cell modes, and coupling with separation techniques, as well as the potential of laser ablation as a spatially resolved sample introduction approach, are explored and discussed. Moreover, this review addresses limitations, novel trends, and perspectives in the field of nanotoxicology, particularly concerning NP internalization and pathways. These processes encompass cellular uptake and quantification, localization, translocation to other cell compartments, and biological transformations. By leveraging the capabilities of ICP-MS, researchers can gain deeper insights into the behaviour and effects of NPs, which can pave the way for safer and more responsible use of these materials.

**Graphical abstract:**

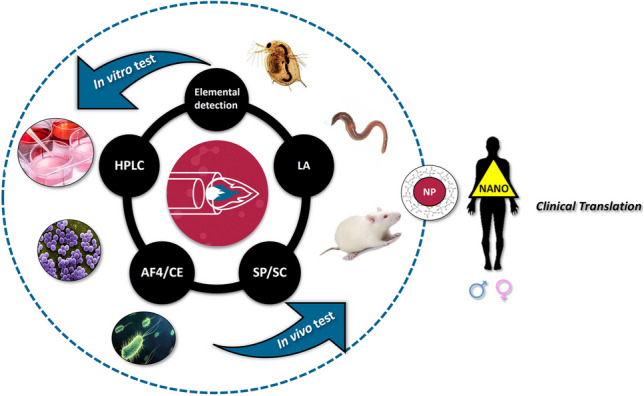

## Introduction

Metallic or metal oxides nanoparticles (NPs) have attracted great interest in different fields due to their exceptional physicochemical properties, such as large surface area, optical activity, chemical reactivity, and mechanical strength [1]. However, the increasing use of these nanomaterials has been accompanied by a growing concern about their safety and impact on human health [2]. Up to now, there is no definitive evidence for their adverse effects, and the current knowledge about the fate and behaviour of NPs in biological systems is still limited. Thus, the study of NP bio-interactions will be crucial for the understanding of their possible risks [3]. Classical toxicological studies are mainly focused on the effects, but the comprehension of the underlying processes is scarce. The assessment of NP toxicity implies the investigation of NP-cell interactions, including cellular uptake, intracellular transport, localization or effects on cell lines, tissues, and organs, among others [4, 5]. Moreover, these NPs can suffer different transformations (i.e. formation of protein corona, ion release) when they are in contact with a biological media [6]. For this reason, a wide variety of instrumental techniques and methodologies enabling the detection, characterization, and quantification of NPs, their degradation products or modified structures in complex matrices, is required to accomplish and achieve a proper interpretation of these nanotoxicological assays.

Traditional approaches for NP characterization, such as microscopic (e.g. transmission electron microscopy (TEM), scanning electron microscopy (SEM) or atomic force microscopy (AFM)) or optical (e.g. dynamic light scattering (DLS) or ultraviolet-visible (UV-vis) spectroscopy) techniques, are mainly focused on physical parameters such as size (size distribution), form, or state of aggregation. But a full analytical information in terms of chemical composition and quantification as well as the monitoring of NP transformations is also necessary to achieve a complete understanding of NP pathways in biological systems. In this sense, mass spectrometry and, more concretely, inductively coupled plasma mass spectrometry (ICP-MS) offers noteworthy capabilities for NPs based on its high sensitivity for multi-elemental and multi-isotopic detection and the possibility of hyphenation with different separation techniques [7]. Accordingly, ICP-MS-based approaches allow to obtain valuable and complementary information about NPs and their potential changes during biological interactions which go from chemical composition, NP core size, and concentration to alterations in hydrodynamic size or information about localization in tissues or dissolution processes as summarized in Table 1.

**Table 1:** Summary of information provided by ICP-MS-based techniques commonly used for NP analysis

Technique	Application	Analytical information
Conventional ICP-MS operation mode	Elemental quantification and identification	- NP identification - Total mass concentration of elemental NP core
Single particle	Sizing and counting (geometry known; mathematical models based on Poisson’s statistics)	- NP identification - NP core size - Size distribution - Particle number concentration - Particle mass-based concentration - Mass concentration of ionic forms
Single cell	Sizing and counting	- NP identification - Number of cell-associated NP - Cell-metal uptake
Laser ablation	(Bio)imaging	- Cell-metal uptake - Cellular localization - Translocation of NPs - Particle mass-based concentration
Hyphenated techniques (HPLC, AF4, CE)	Nano-biointeraction/biotransformation monitoring	- NP hydrodynamic volume/diameter - Size distribution - Particle-mass based concentration - Mass concentration of ionic forms - Aggregation or agglomeration status (not in CE)

*AF4*, asymmetric flow field-flow fractionation; *CE*, capillary electrophoresis; *HPLC*, high performance liquid chromatography

The role of ICP-MS for the study of NPs has been addressed in previous reviews [4, 7–10]. A global overview of mass spectrometry for the characterization of inorganic NPs [7] or materials at the nanoscale [8] has already been presented, but without specifically focusing on ICP-MS and the biological field. Modern methodologies and techniques used for the study of NP-cell interactions and cellular processing including ICP-MS, among others, have also been revised for NPs in general [9] or with gold nanoparticles (AuNPs) as a case study [4]. Galazzi et al*.* [10] reviewed the considerable increase of the application of ICP-MS for the assessment of NPs in last decades including a brief discussion about studies of NP effects mainly focused on plants. However, it is worthwhile to further study and critically evaluate the importance of various ICP-MS modalities to provide relevant information for a proper interpretation of in vitro and in vivo toxicological assays. The possibilities of conventional analysis, single particle (SP) or single cell (SC) modes, or hyphenated separation techniques as well as the potential of laser ablation (LA) for imaging are critically revised in the present review with focus on toxicological applications. Recent advances in the nanotoxicology field related to the knowledge of particle internalization and cellular processing of NPs (i.e. NP uptake and quantification, localization, changes in the chemical state or size) are addressed. Novel trends and perspectives are also discussed to provide a global overview of ICP-MS-based platforms contributions and future developments.

## Assessment of metallic nanoparticles fate and pathways in toxicological studies by means of ICP-MS-based platforms

The knowledge of the mechanisms of NP internalization as well as the quantification of NP uptake by cells is of major interest for biomedical applications. Numerous unanswered questions persist regarding how the NP properties influence the initial interaction at the cell surface, the potential recognition by cell receptors, and the subsequent internalization mechanisms [11]. There are also remaining difficulties for the proper understanding of NP internalization related to the adequate discrimination between the NPs that have been really internalized and those that have only been adsorbed into the cell surface. Accordingly, the cellular uptake and distribution of NPs has already been extensively studied and revised [11–15], even when the detection and localization of NPs within cells is still extremely challenging.

### Qualitative information: NP localization

**Fig. 1:**
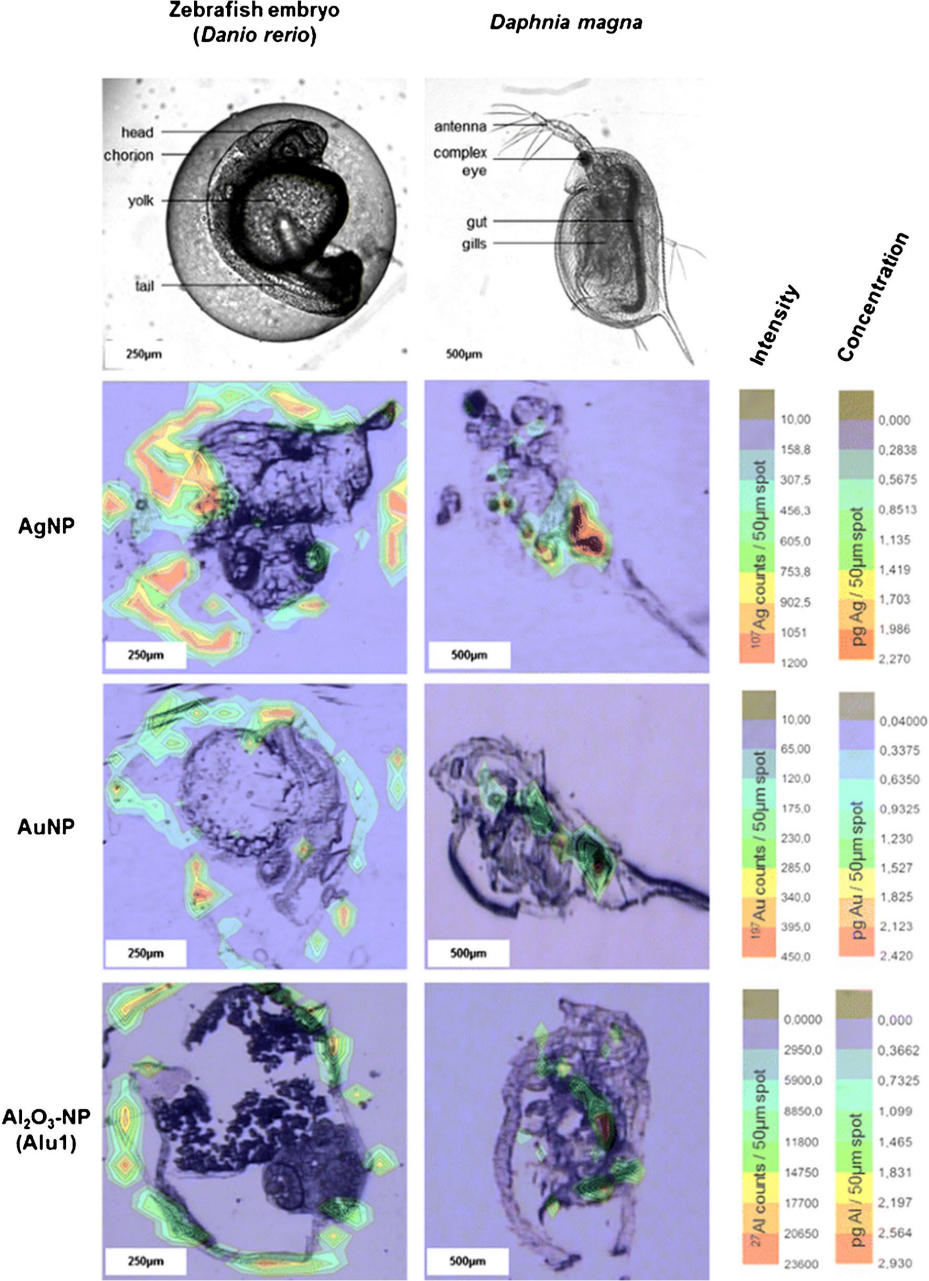
Visualization of AgNPs, AuNPs, and Al_*2*_O_3_NPs distribution in terms of signal intensity for *Danio renio* and *Daphnia magna* via LA-ICP-MS through a 50 μm spot ablation of 40-μm-thick organism sections. (Reprinted with permission from Böhme et al. (2015), ^©^2015, Springer)

The localization of NPs at their sites of interaction with biological media and tissues is also a key point for a thorough interpretation of toxicological studies. Different approaches including traditional microscopic techniques (i.e. TEM and SEM) as well as new microscopic or spectroscopic developments can be used for NP visualization, but none of them accomplish all the needed technical requirements in terms of optical resolution, sensitivity, spatial information for bioimaging, or applicability for specific NPs [16]. In this context, LA in its combination with ICP-MS has emerged as a compelling spatially resolved sample introduction approach for studies on NP distribution and/or possible accumulation in toxicological assays [10], which provides relevant spatial information with little sample preparation even for solid samples [10, 17]. Thus, LA-ICP-MS is an ideal option for elemental mapping of single cells and tissues becoming an interesting alternative to other techniques where samples must be liquid and no spatial information can be obtained. This technique has been widely employed in the biological field for elemental monitoring, but its potential to provide qualitative information of NP localization has been less explored so far [18]. LA-ICP-MS has been mainly used for NP qualitative imaging and mapping using vegetal tissues such as onion cells, radish, or sweet basil leaves as a case study [19]. Nevertheless, it was also employed in in vitro assays. Thus, Hsiao et al*.* [20] combined various ICP-MS-based platforms, including LA, to investigate the localization of 50 and 75 nm AgNPs and 7 and 20 nm titanium dioxide NPs (TiO_2_NPs) in Neuro-2a mouse neuroblastoma cells. In the case of TiO_2_NPs, the smaller particles (7 nm) penetrate easily into cells. It was also demonstrated that the 50 nm AgNPs were located within cells whereas a distinct scenario was found for the 75 nm particles which were adsorbed onto the cell membranes. Regarding its application for the assessment of NPs in in vivo assays, LA-ICP-MS was employed for the qualitative mapping of 13 nm AuNPs, 21 nm AgNPs, and 14, 111, and 750 nm Al_2_O_3_NPs in zebrafish (*Danio rerio*) and crustacean (*Daphnia magna)* embryos [21]. The images and signal intensities for both model organisms are shown in Fig. 1. A heterogeneous distribution of these nano-sized particles was observed. Respect to *D. rerio*, most NPs are accumulated at the outermost membrane surrounding the embryo called chorion. Regarding to *D. magna*, the images showed a NP uptake specially in the gut followed by the gill and eye tissues in a minor proportion.

With respect to the investigation using classical animal models, Elci et al*.* [22–24] studied the biodistribution of 2 nm functionalized AuNPs administered intravenously to different laboratory (Balb/c) mice tissues as spleen, liver, lung, and kidney. LA-ICP-MS images revealed that the NP surface charge influence in the interaction with the different organs [23]. Moreover, they demonstrated that NP stability differs considerably in the different organs which influences the stability profiles for NPs in in vitro and in vivo assays [24], confirming that conclusions of in vitro studies cannot always be directly transferred to in vivo conditions. Reifschneider et al*.* [25] discussed and compared the employment of different techniques with high sensitivity and capacity for spatially resolved element analysis (LA-ICP-MS and synchrotron radiation) for the study of 50 nm AgNPs in female Wistar rat lung tissue. It was demonstrated that the information provided by these imaging techniques enable to obtain a reliable assessment of the NPs distribution and pathways at single cell level.

### Quantitative information: NP cellular uptake

ICP-MS-based approaches also become a good alternative for the quantification of cellular uptake. A collection of in vitro and in vivo toxicological assays conducted in the last decade where the uptake of different NPs has been investigated and quantified using ICP-MS-based systems is presented in Tables 2 and 3.

**Table 2:** Summary of studies using ICP-MS-based systems for the assessment of NP cellular uptake quantification through in vitro toxicological studies

NP	Shape/size (nm)/functionalization	Cell line	Sample preparation	Technique	Reference
AgNP	Spherical/15 nm/citrate	HepG2	Acid digestion/alkaline extraction	ICP-MS & AF4	[26]
	Spherical/50, 75 nm/citrate	Neuro-2a	Acid digestion/alkaline extraction	ICP-MS, SP & LA	[20]
	Spherical/50 nm /citrate	THP-1	Acid digestion/UPW dilution	ICP-MS & SC	[27]
	Spherical/20, 75 nm/citrate	ARPE-19	Acid digestion/alkaline extraction	ICP-MS & SP	[28]
	Spherical/20 nm	16HBE	Enzymatic extraction	LA-ICP-MS	[29]
	Spherical/50 nm/citrate	NIH-3T3	Acid digestion/enzymatic extraction	ICP-MS & LA	[30]
AuNP	Hexapods, rods & cages/hexapods (25 nm), rods (36 nm), 47 nm (cages)	MDA-MB-435	Enzymatic extraction	ICP-MS	[31]
	Rod/10–40 nm	Vero, MRC-5 & NIH3T3	Acid digestion	ICP-MS	[32]
	Spherical/2, 6, 10, 16 nm/citrate	MCF-7	Acid digestion	ICP-MS	[33]
	Spherical/graphene oxide/Au (13 nm)	A549 & HepG2	Enzymatic extraction	ICP-MS	[34]
	Spherical/4–5 nm	HeLa	Acid digestion	ICP-MS	[35]
	Rod/PEG	PC-3	Acid digestion	ICP-MS	[36]
	Spherical/15, 50, 100 nm/citrate	Caco-2	Acid digestion	ICP-MS	[37]
	Rod/88 nm	Kupffer	Acid digestion	ICP-MS	[38]
	Spherical/10 nm/citrate	HeLa	Acid digestion/dilution with culture medium	ICP-MS & HPLC	[39]
	Spherical/40, 80 nm/PEG	HPTC	Acid digestion	ICP-MS	[40]
	12 nm	MCF-7	Acid digestion/enzymatic extraction	ICP-MS & SC	[41]
	Spherical/32 nm/PEG	MDA-MB-321 & T-47D	Acid digestion/alkaline extraction	ICP-MS & SP	[42]
	Spherical/15, 30, 60 nm/citrate	HeLa	Acid digestion/enzymatic extraction	ICP-MS & SC	[43]
	Spherical/20, 50 nm	MCF-7 & MCF-10a	Acid digestion/enzymatic extraction	ICP-MS, SP, HPLC & CE	[44]
	Spherical/20–100 nm	Melanoma cancer cells	Acid digestion	ICP-MS	[45]
	10, 30, 60 nm/citrate	HT-29 & HepG2	Acid digestion/enzymatic extraction	ICP-MS & HPLC	[46]
	Spherical/17, 19 nm	HepG2, HeLa	Acid digestion	ICP-MS	[47]
	Spherical/30 nm (NIST RM 8012)	Raw 264.7	Acid digestion/enzymatic extraction	ICP-MS & LA	[48]
FePtNP	7 nm	HepG2	Acid digestion/enzymatic extraction	ICP-MS & SC	[49]
PtNP	Spherical/1–21 nm/PVP	Neuro-2a	Acid digestion	ICP-MS	[50]
	Spherical/4–9 nm/PVP	EPC & BF-2	Acid digestion	ICP-MS	[51]
Ag/NiO—Ag_2_O/NiO/ ZnO nano-composites	2, 4, 6, 32 nm	Vero	Acid digestion	ICP-MS	[52]
Al_2_O_3_	Spherical to irregular shape/14, 111, 750 nm	HaCaT & A549	Acid digestion/enzymatic extraction	ICP-MS & LA	[53]
Fe_2_O_3_	Spherical/3–4 nm/tartaric, and adipic acids	Caco-2 & HT-29	Acid digestion/enzymatic extraction	ICP-MS & HPLC	[54]
	Spherical/5, 10 nm	HepG2, HeLa	Acid digestion	ICP-MS	[47]
SPIO-AuNP	Quasi-spherical/6, 7 nm	PC-12	Acid digestion	ICP-MS	[55]
SiO_2_	Spherical/25, 28 nm	HepG2, HeLa	Acid digestion	ICP-MS	[47]
TiO_2_	Spherical/7, 20 nm /citrate	Neuro-2a	Acid digestion/fixation (PFA-PBS)	ICP-MS, SP & LA	[20]
	Spherical/13 nm/citrate	A549	Acid digestion	ICP-MS	[56]
	Spherical/1, 10 nm	HepG2, HeLa	Acid digestion	ICP-MS	[47]

Functionalization: *PEG*, polyethylene glycol; *PVP*, polyvinylpyrrolidone. Cell lines: *16HBE*, human bronchial epithelial; *A549*, adenocarcinomic human alveolar basal epithelial; *ARPE-19*, human retinal pigment epithelial; *BF-2*, Bluegill fibroblast; *Caco-2*, human colorectal adenocarcinoma; *EPC*, epithelioma papulosum cyprini*; HaCaT*, aneuploid immortal keratinocyte; *HeLa*, human cervical adenocarcinoma; *HepG2*, human hepatocyte carcinoma; *HPTC*, human papillary thyroid; *HT29*, human colon cancer; *Kupffer*, human liver; *MCF-7*, human breast cancer; *MDA-MB*, human breast cancer adenocarcinoma; *MRC-5*, human foetal lung fibroblast; *Neuro-2a*, mouse neuroblastoma; *NIH3T3*, mouse embryonic fibroblast; *PC-3*, human prostatic; *PC-12*, adrenal phaeochromocytoma; *THP-1*, human leukaemia monocytic; *T-47D*, human breast cancer; *Vero*, kidney epithelial. NPs: *SPIO*, superparamagnetic iron oxide

**Table 3:** Summary of studies using ICP-MS-based systems for the assessment of NP uptake through in vivo toxicological studies

NP	Shape/size (nm)/functionalization	Organism	Sample preparation	Technique	Reference
AgNP	30, 70/PVP-coated and non-coated	Earthworm (*Lumbriculus variegatus*)	Sonication with UPW	ICP-MS, AF4 & SP	[57]
	Spherical/60, 100 nm/PVP	*Crustacean* (*Daphnia magna*) & earthworm (*Lumbriculus variegatus*)	Alkaline extraction	SP-ICP-MS	[58]
	20 nm/PVP or citrate	Cyprinid fish (*Pimephales promelas*)	Acid digestion/sonication with UPW	ICP-MS & AF4	[59]
	Spherical/21 nm	Crustacean (*Daphnia magna*)* &* zebrafish (*Danio rerio*)	Acid digestion/fixation (PFA-PBS)	ICP-MS & LA	[21]
	Spherical/25, 75 nm PVP	Nematode (*C. elegans*)	Acid digestion	ICP-MS	[60]
	Spherical/50 nm/citrate, PVP, silicate, bPEI	Trout (Oncorhynchus mykiss)	Acid digestion	ICP-MS	[61]
	Variable shape/26 nm	Zebrafish (*Danio rerio*)	Acid digestion	ICP-MS	[62]
	Spherical/20, 100 nm/CIT, PVP	Zebrafish (*Danio rerio*)	Acid digestion	ICP-MS	[63]
	20 nm	Mice (CD-1)	Alkaline extraction	SP-ICP-MS	[64]
	Spherical/50 nm	Wistar rat lung tissue	Acid digestion / Fixation (PFA-PBS)	ICP-MS & LA	[25]
AuNP	Spherical/100 nm/PVP	Crustacean (*Daphnia magna*) & earthworm (*Lumbriculus variegatus*)	Alkaline extraction	SP-ICP-MS	[58]
	13 nm	Crustacean (*Daphnia magna) &* zebrafish *(Danio rerio)*	Acid digestion/fixation (PFA-PBS)	ICP-MS & SC	[21]
	2 nm	Mice (female Balb/c)	Acid digestion	ICP-MS	[22]
	Spherical/30, 60 nm (NIST 8012, NIST 8013)/citrate	Nematode (*C. elegans)*	Acid digestion	ICP-MS	[65]
	Spherical/80, 100, 150 nm citrate	Nematode (*C. elegans),* bacteria *(E. coli)*	Alkaline extraction	SP-ICP-MS	[66]
	10 nm	Wistar rat liver & spleen	Enzymatic extraction	HPLC-ICP-MS	[67]
	40 nm	Rat liver	Sonication with lysis buffer	SP & HPLC-ICP-MS	[68]
	Spherical 10, 60, 100 nm Rods	Crustacean (*Daphnia magna) &* zebrafish *(Danio rerio)*	Alkaline extraction	SP & SC-ICP-MS	[69]
	10, 30, 60 nm/citrate	Wistar rats tissues	Acid digestion/enzymatic extraction	HPLC-ICP-MS	[46]
Al_2_O_3_	14, 111, 750 nm	Crustacean (*Daphnia magna*)* &* zebrafish (*Danio rerio*)	Acid digestion/fixation (PFA-PBS)	ICP-MS & LA	[21]
Fe_2_O_3_	< 4 nm/tartaric, and adipic acids	Wistar rat tissues	Acid digestion/enzymatic extraction	HPLC-ICP-MS	[70]
TiO_2_	< 100 nm	Mussels (*Mytilus galloprovincialis*)	Acid digestion/alkaline extraction	ICP-MS & SP	[71]
	Spherical/21 nm	Zebrafish	Acid digestion/enzymatic extraction	ICP-MS & SP	[72]
	Spherical/ < 25 nm	Rat organs	Acid digestion/enzymatic extraction	ICP-MS & SP	[73]
ZnO	Spherical/30 nm	Canned seafood	Acid digestion/enzymatic extraction	ICP-MS & SP	[74]

**Fig. 2:**
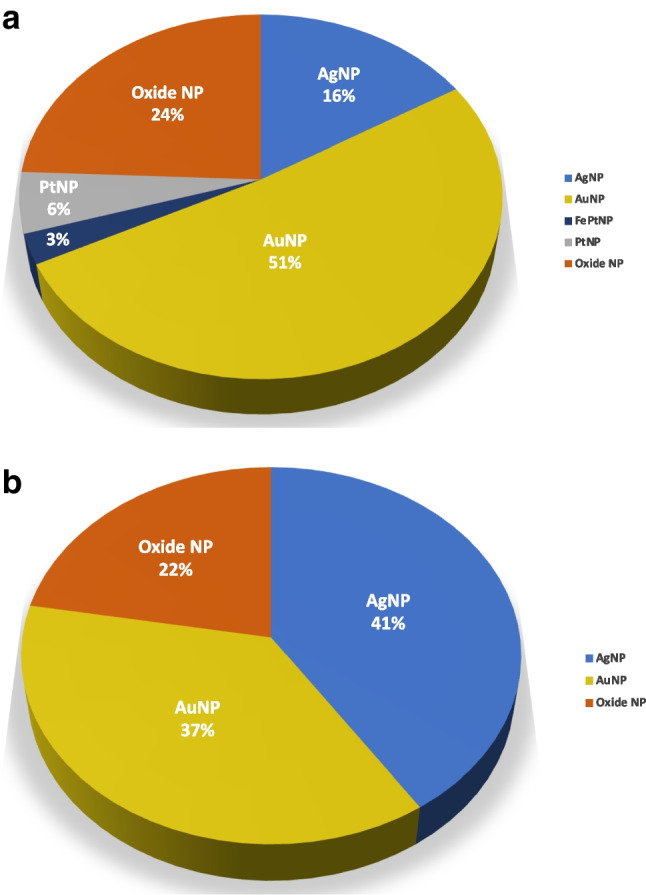
Distribution of scientific works reporting **a** in vitro or **b** in vivo toxicological studies using ICP-MS-based systems for the assessment of NP cellular uptake as a function of NP core

It should be noted that the type of NP mostly investigated differs between in vitro and in vivo experiments (Fig. 2). Thus, AuNPs have been by far the predominant targets in in vitro assays up to the moment, although the uptake of AgNPs and PtNPs, as well as some oxide NPs, mainly alumina, silica, and titania, have also been tackled (Fig. 2 and Table 2). NP oxides (including those of Al, Fe, Ti, or Zn) have also been studied under in vivo conditions, although internalization of AuNPs and AgNPs have been mostly assessed (Table 3). Regarding to shape, the spherical where the preferred NPs in all cases. It is also remarkable that in vitro cellular quantitative uptake studies (Table 2) have been mainly focused on cancer cell lines including human breast (MCF-7, MDA-MB, T-47D), cervical (HeLa), colon (HT29), colorectal (Caco-2), liver (HepG2), or prostate (PC-3) carcinomas, among others. The behaviour of non-cancer cell lines, such as endothelial, epithelial, and fibroblast cells, which are three of the most common cell types in the composition of most organs, has also been monitored (Table 2). Concerning in vivo assays (Table 3), aquatic organisms (i.e. algae, crustacean, zebrafish) account for about 40% of the reviewed works, whereas terrestrial invertebrates (earthworms or nematodes) and model animals (mice (CD-1) or rats (Wistar)) appear in about 60% of the publications. Moreover, alternative models based on the use of terrestrial invertebrates, zebrafish, or crustacean are replacing the traditional application of higher animals in in vitro assays [94]. Thus, the nematode *C. elegans*, the zebrafish *Dario renio,* or the crustacean *Daphnia magna* have become valuable tools and are largely used for the investigation of NP toxicity [21, 58, 60, 62, 63, 65, 66, 69].

Regarding the test conditions, as a rule of thumb, in vitro toxicological studies involved the incubation of cells with NPs during different times (usually at 37 °C and up to 24 h) and a subsequent separation of supernatants and cells before analysis, whereas in in vivo experiments, the conditions were extremely variable due to the wide diversity of organisms employed. Moreover, the elemental characterization of NPs usually involves a careful sample pre-treatment, which plays a key role in these quantitative analyses, and it is by far the more troublesome step in the analytical process.

The diversity of NP properties and complexity of toxicological matrices implies to handle the sample before the ICP-MS measurements in their different modalities preserving the integrity of the original NPs. It should be then considered that the procedures applied for extraction, separation, and/or preconcentration of NPs from complex samples are dependent not only on the target analyte and the analytical technique but also on the sample matrix and that the potential interferences and difficulties in this step must be addressed to achieve a convenient harmonization of the results. Thus, the requirements of sample preparation methods for the determination of NP total element content or characterization in terms of size and/or concentration differ considerably. Total elemental analysis of NPs is mainly based on acid digestion with heating, usually performed in a microwave oven. Nitric acid used to be the chosen reagent, although it has also been employed in combination with other acids (*aqua regia*) or hydrogen peroxide [75]. Using these conditions, the information about NP itself is lost because the NP is transformed into its ionic form. Thus, to assure the NP integrity and enable the analysis of NPs as individual entities, alkaline and/or enzymatic extractions have been extensively employed in the toxicological assays (Tables 2 and 3). Concerning the procedures for clean-up and purification, the evaporation, centrifugation, and filtration are the most common alternatives, although they can be time-consuming and induce aggregation. Among them, centrifugation is generally considered the less perturbing strategy, but the differential settling of particles during this process may also increase the probability of NP colliding and the formation of aggregates. Moreover, other additional steps such as complexation of interfering ions or removal of proteins or fats cannot be discarded [75, 76].

**Fig. 3:**
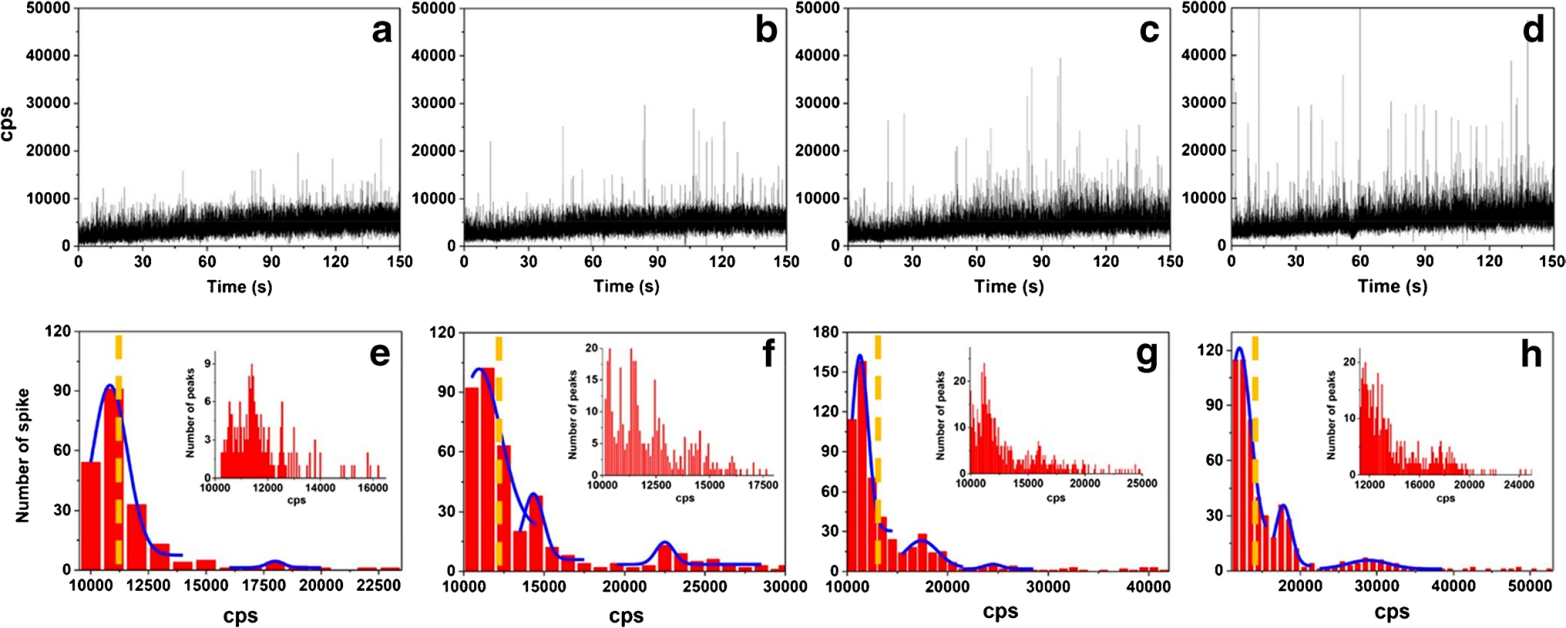
Temporal profile (**a**–**d**) and frequency histograms (**e**–**h**) of ^197^Au in MCF-7 cells by incubating in a medium containing 1.0 μmol L^−1^ AuNPs for 1, 2, 4, and 8 h. (Reprinted with permission from Wei et al. (2018), ^©^2018 Springer)

With respect to instrumentation, toxicological assays usually involve, as a first step, the quantification of the NP total elemental content conducted by conventional ICP-MS after digestion (Tables 2 and 3). The advantages of ICP-MS for this total quantification are related to the high sensitivity and robust elemental detection offered by this detector. Nevertheless, other approaches such as SP/SC-ICP-MS or hyphenated systems with AF4, CE, or HPLC have been extensively used to study the uptake and track the NP dynamics and fate in the biological media and organisms investigated using mild sample preparation conditions (Tables 2 and 3). The precise determination of NP number concentration is a relevant information for the characterization of NPs in quantitative terms. This number concentration cannot be directly obtained by conventional ICP-MS analysis, but its operation in SP mode stands out as an interesting alternative for particle number determination limited to spherical NPs. The information about NP number concentration after cellular uptake may be accompanied by the mass-based concentration of both NPs and dissolved species as well as NP size, demonstrating the great potential of this analytical tool for the simultaneous NP counting and sizing at low concentrations, which enables its wide application in toxicological assays (Tables 2 and 3). Based on the same principle of SP-ICP-MS but considering the individual detection of cells emerges the concept of SC-ICP-MS. This technique allows the quantification of cell metal content, considering that each cell is an individual (bio)entity [77, 78]. The correct detection of the signal generated by individual cells may be hampered by high backgrounds, interferences, or high concentration of ionic ions or particles, especially in the case of complex media. Thus, an adequate cell isolation from the media and further resuspension in a fresh medium or isotonic solution can be required in some cases for the determination of cell metal uptake [18]. For instance, SC-ICP-MS was used by Wei et al*.* [41] who follow the uptake of AuNPs within MCF-7 cells and observed a large intercellular heterogeneity in this uptake (130–584 AuNPs cell^−1^) (Fig. 3)**.** LA-ICP-MS can also be employed for NP uptake quantification enabling the analysis of a smaller number of cells than SC-ICP-MS where about thousands of single cells per sample are needed [29]. However, the assessment of NP elemental concentrations in single cells by LA-ICP-MS is challenging especially due to the limitations related to the calibration method. As it operates with solid samples, their structure could change during the ablation, and it would make no possible to discern between the NP and the matrix signals [19]. Moreover, the lack of suitable standards for calibration and the slow analytical throughput hinders its application in quantitative elemental analysis. Different calibration approaches (i.e. via matrix matched calibration, dried nanodispersion droplet, spiked slices, or isotope dilution) have already been tested aiming to achieve more homogenous elemental distributions [19]. But up to now, there is no agreement about the adequate calibration method to achieve reliable quantification. In addition, this technique also exhibits some drawbacks concerning the sensitivity which is considerably higher than other laser-based techniques (i.e. laser-induced breakdown spectroscopy (LIBS)) but still in the mg L^−1^ level [22]. Moreover, it is worth noting the importance to control the crater size related to the size of the cell or the need of implementation of wash-out systems to improve the laser scan speed [79]. Different separation techniques interfaced with ICP-MS have also demonstrated outstanding capabilities for NPs quantification and speciation although they are mainly employed for the monitoring of the interactions between NPs and biomolecules which result in NP transformations as it will be discussed in the next section.

### Monitoring NP transformations

Studies where ICP-MS-based techniques have been employed to follow NP transformations in in vitro and in vivo toxicological assays are summarized in Table 4**.**

**Table 4:** Summary of studies using ICP-MS-based systems for the assessment of NP transformations in in vitro and in vivo toxicological studies

Technique	NP	Shape/size (nm)	Media/cell line/organism	Transformations	Reference
In vitro assays					
AF4-ICP-MS	Ag	Spherical/15	DMEM-high glucose, HepG2	Protein corona formation, oxidation	[26]
	Au	Spherical/10, 30, 40	DMEM-high glucose	Protein corona formation, oxidation	[80]
	Fe_2_O_3_	Spherical/27–30	Rat blood plasma, cell fractions	Changes in hydrodynamic size	[81]
CE-ICP-MS	Au	Spherical/20, 50	MCF-7, MCF-10a	Protein corona formation	[44]
	SP-Fe_2_O_3_	Spherical/15, 20	Human blood serum	Protein corona formation	[82]
HPLC-ICP-MS	Au	Spherical/10	DMEM-high glucose, HeLa	Protein corona formation, oxidation	[39]
	Fe_2_O_3_	Spherical/5.9	A2780	Protein corona formation	[83]
	Pt	Spherical/5, 30	DMEM, and RPMI-1640	Protein corona formation, oxidation	[84]
SP-ICP-MS	Ag	Spherical/20, 75	ARPE-19	Changes in NP core size	[28]
	Au	Spherical/40, 60, 80	DMEM-high glucose	No differences in NP size and size distribution	[85]
	Au	Spherical/20, 50	MCF-7	Changes in NP core size	[44]
	Fe_2_O_3_	Spherical/27–30	Rat blood plasma, cell fractions	Changes in NP size in plasma and no changes in cells	[81]
	Ag, TiO_2_	Spherical/50, 75 (Ag); 7, 20 (TiO_2_)	Neuro-2a	Aggregation	[20]
In vivo assays					
AF4-ICP-MS	Ag	Spherical/20	*Pimephales promelas*	Aggregation	[59]
	Ag	Spherical/30, 70	*Lumbriculus variegatus*	Changes in hydrodynamic size, dissolution	[57]
SP-ICP-MS	Ag	Spherical/30, 70	*Lumbriculus variegatus*	Changes in NP core size, dissolution	[57]
	Au	Spherical/30, 60	*Caenorhabditis elegans*	Changes in NP core size	[65]
	Au	Spherical/60	Rat spleen	No differences in NP size and size distribution	[86]
	Au	Spherical/40	Rat liver	No differences in NP size and size distribution, dissolution	[68]
	TiO_2_	Spherical/ < 100	*Mytilus galloprovincialis*	NP formation in vivo	[71]

**Fig. 4:**
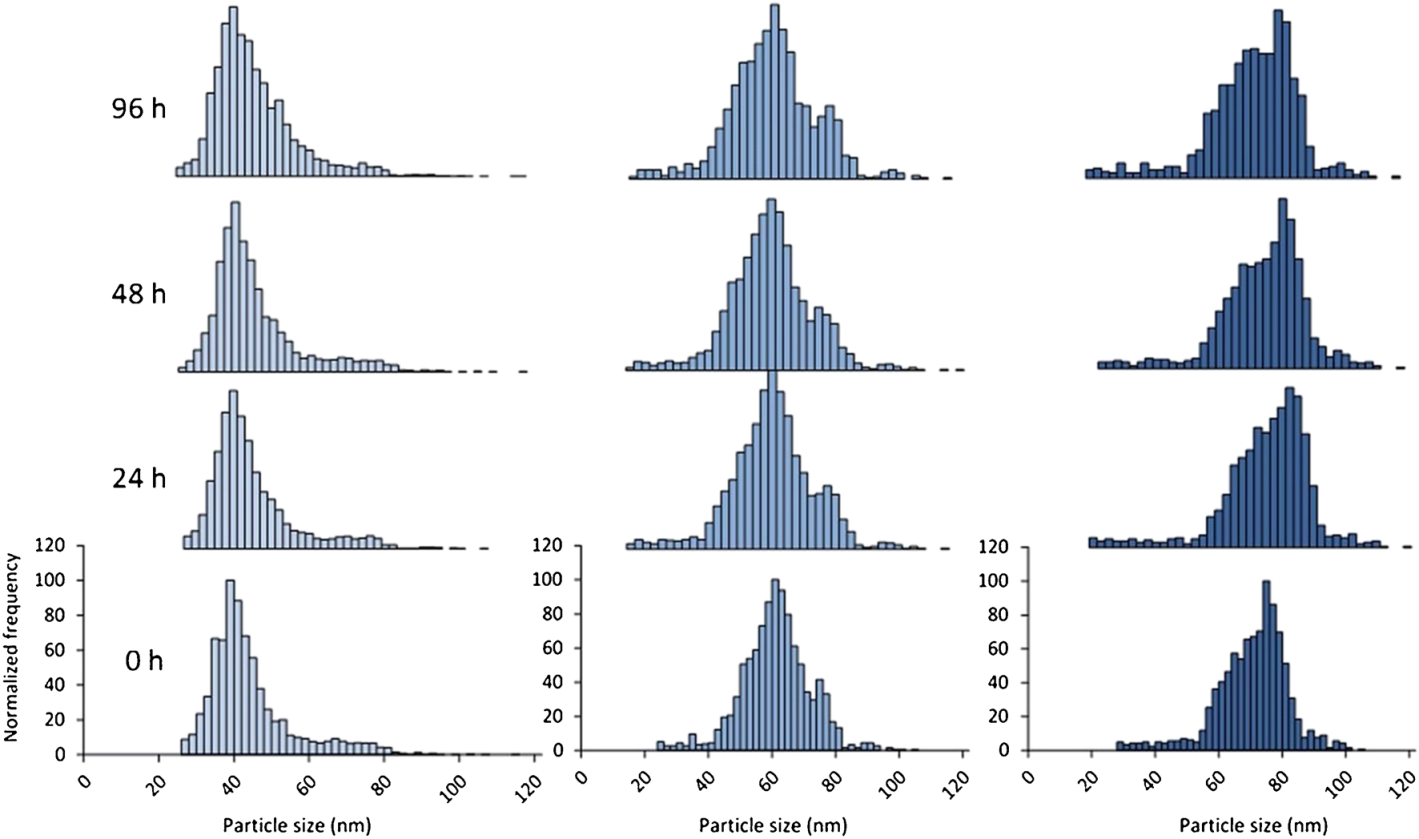
Particle size distribution histograms by the optimized SP-ICP-MS approach related to the effect of incubation time (0, 24, 48, and 96 h) for the different AuNPs: 40 nm (light blue), 60 nm (medium blue), and 80 nm (dark blue) upon the exposure of DMEM-high glucose (supplemented with 10% FBS and antibiotics). (Reprinted with permission from Fernández-Trujillo et al. (2021a) ^©^2021, The Royal Society Chemistry)

NP transformations related to changes in particle size and size distribution can be followed using ICP-MS in SP mode. In the case of in vitro tests, several works were devoted to the study of NP transformations in different types of cancerous cell lines. For instance, Kruszewska et al*.* [44] tracked the changes in size of AuNPs using different ICP-MS-based techniques in MCF-7 cancer cell lines. SP-ICP-MS was used to confirm the stability of NP, since particle size scarcely increased, and the absence of ionic gold forms inside the cells was observed. The crucial role of the culture medium in the adequate interpretation of in vitro studies has also been explored using AuNPs incubated in Dulbecco’s Modified Eagle Medium (DMEM-high glucose) [84]. Although it can be expected that AuNPs were coated by species present in the culture media, no changes in terms of particle size were observed up to 96 h of exposure time at 37 °C for the different sized-AuNP studied (40, 60, and 80 nm). This observation confirms that the potential adsorption of matrix components over the NP surface produces changes in the hydrodynamic diameter, but not in the NP core size. However, the incubation time influenced the particle number and mass concentrations, with a notable decrease in the case of 80 nm AuNPs (Fig. 4). NP size transformations in in vivo bioassays have also been followed using the SP-ICP-MS capabilities. Thus, Loeschner et al*.* [86] and Álvarez-Fernández García et al*.* [68] studied the possible AuNP transformations in different rat tissues, but no changes in NP size were found. Concerning the interactions of NP with model organisms, both *Lumbriculus variegatus* [57] or *Caenorhabditis elegans* [65] have been investigated, reporting an increase in AgNP or AuNP sizes, respectively.

The interactions of NPs with the biological media can trigger process such the formation of protein corona which can cause changes in the NP hydrodynamic size. This phenomenon involves the genesis of a dynamic multi-layered structure which incorporates two parts: hard and soft corona. The soft corona is the outer layer and comprises proteins with low affinity to NP surface, so it suffers dynamic and reversible exchanges dependent on the biological media conditions. Regarding to the hard corona, it is the inner layer formed by high affinity proteins, which confers more stability. The different nature of both structures influences on the way of isolation although centrifugation is the most widely used method for the isolation of NP-protein corona complexes [6]. To understand the fate of these nano-sized complexes in biological systems, or their potential toxicological effects, an accurate and reliable characterization of protein corona composition is required. For this purpose, a combination of multiple analytical methodologies (i.e. elemental and molecular MS or other spectroscopic techniques) offering complementary information appears indispensable. Main challenges persist in the effective quantification of the diverse proteins within the corona. In this context, ICP-MS turns up as a complementary tool capable of providing accuracy and robustness in quantitative results and workflows due to its species-independent nature, in contrast to common molecular MS approaches for protein quantification such as electrospray (ESI-MS). However, ICP-MS itself cannot provide a comprehensive picture of the diverse NP-protein corona populations present in the sample. Thus, the use of separation techniques, such as HPLC, AF4, and CE hyphenated to ICP-MS, appears necessary to provide a comprehensive review of the protein corona complexes. Nonetheless, it is important to keep on mind that these ICP-MS approaches only provide an absolute protein quantification and molecular MS will be required to achieve a detailed characterization of the protein corona including the information about the identity of the proteins (for instance, by using LC coupled to mass spectroscopy (LC-MS/MS)) [87, 88].

**Fig. 5:**
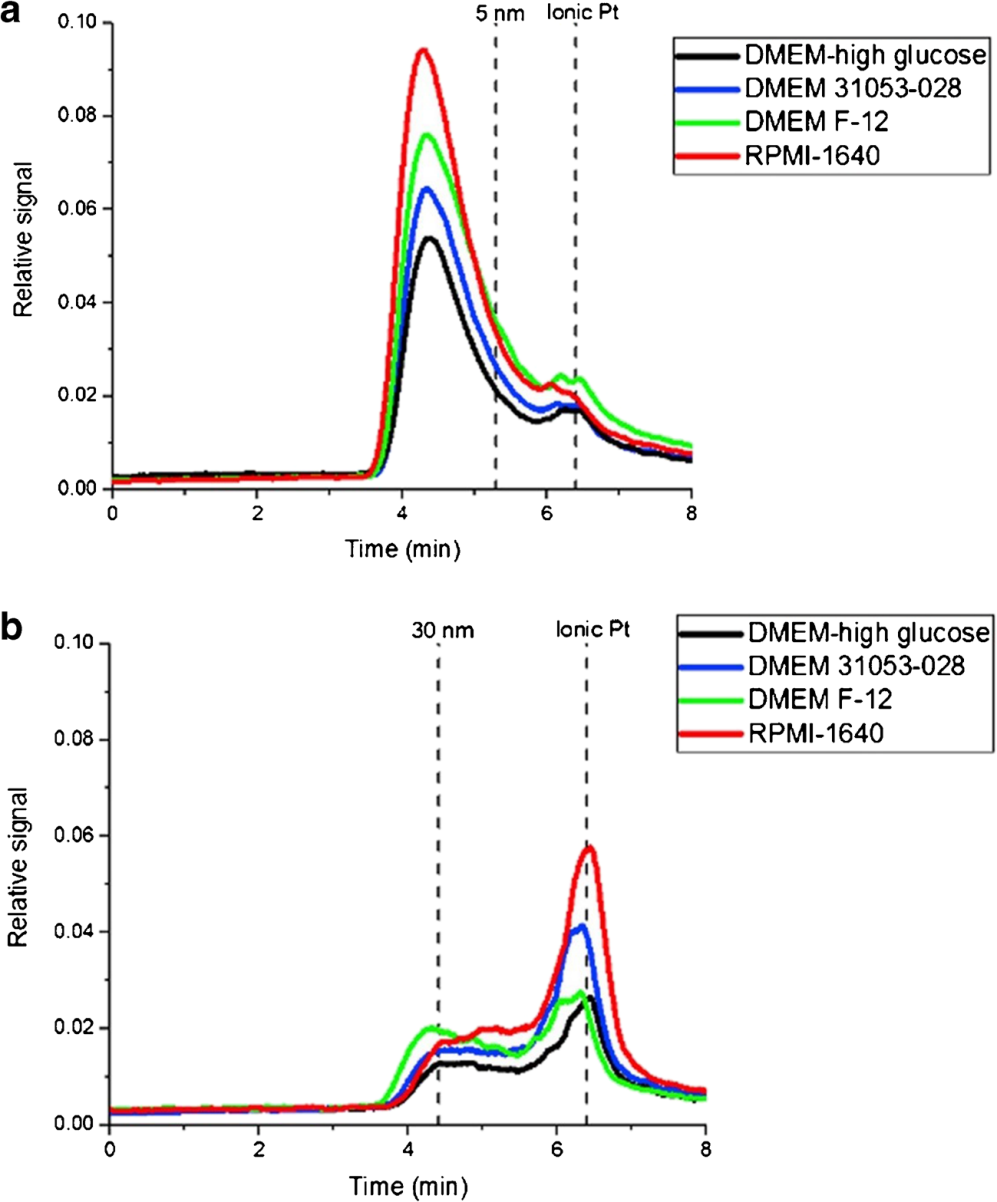
HPLC-ICP-TQ-MS chromatograms at 0 h of incubation time of **a** 5 nm and **b** 30 nm PtNPs in different biological media including DMEM-high glucose, DMEM F-12, DMEM 31053-028, and RPMI-1640 (supplemented with 10% FBS and antibiotics). Standards in mobile phase are shown as vertical lines for better understanding. (Reprinted with permission from Fernández-Trujillo et al. (2021b), ^©^2021, Elsevier)

Approaches based on chromatographic separation become an attractive option due to their simplicity and cost-effectiveness. Different alternatives including hydrodynamic chromatography (HDC), size exclusion chromatography (SEC), or HPLC can be theoretically considered, although the separation capabilities of HDC and SEC are really poor in comparison to other approaches. Therefore, the preferred chromatographic combination has been HPLC, usually using a C18 reversed-phase column [89], with a separation based on a size-exclusion mechanism related to the hydrodynamic volume so the larger entities would elute first. Helfrich et al*.* were the pioneers in the proposal of reverse-phase chromatography for the separation of NPs using AuNPs as a case study [90, 91]. Regarding the application of this approach in toxicological assays, HPLC-ICP-MS was employed by López-Sanz et al*.* [39] for the characterization and identification of 10 nm AuNPs and dissolved Au species (Au^3+^) in DMEM-high glucose (supplemented with 10% FBS, and antibiotics) and HeLa cells. The results confirm that the interaction between AuNPs and their ionic counterparts in DMEM-high glucose induces the formation of a protein corona that changes their size distribution. Moreover, 10 nm AuNPs exposed to HeLa cells revealed a slight toxic effect. The same behaviour was also observed by Fernández-Trujillo et al*.* [84], which developed a fast and simple strategy based on HPLC-ICP-TQ-MS for the study of 5 and 30 nm PtNPs in different cell culture media (DMEM-high glucose, DMEM-F12, DMEM 31053-028, and Roswell Park Memorial Institute, RPMI-1640 (all supplemented with 10% FBS and antibiotics)) at several incubation times (24, 48, and 96 h). The chromatograms of PtNPs in presence of DMEM-high glucose revealed a displacement to lower retention times and a significant decrease in terms of relative signal intensity for both sizes. These findings can be related to the protein adsorption over the NP surface which produces an increase in the hydrodynamic size. It was also remarkable that the chromatographic profiles for 5 (Fig. 5a) and 30 nm PtNPs (Fig. 5b) were similar in the different biological media tested and that the study of incubation times revealed dynamic changes over time that should be considered. Furthermore, Turiel-Fernández et al*.* [83] employed the HPLC-ICP-MS technique for the characterization of a Fe_2_O_3_NPs cisplatin (IV) prodrug nanoconjugate incubated in different ovarian cancer cell lines. The chromatogram showed a single, and narrow peak at 5.6 min (6–7 nm) demonstrating the formation of the Pt(IV) prodrug-NP conjugate in comparison with the hydrodynamic size before conjugation (5.9 ± 0.9 nm). The use of complementary techniques as DLS (hydrodynamic diameter: 6.6 ± 1.0 nm; ζ -potential: from − 2.5 to 1.08 mV) supported the HPLC-ICP-MS data.

**Fig. 6:**
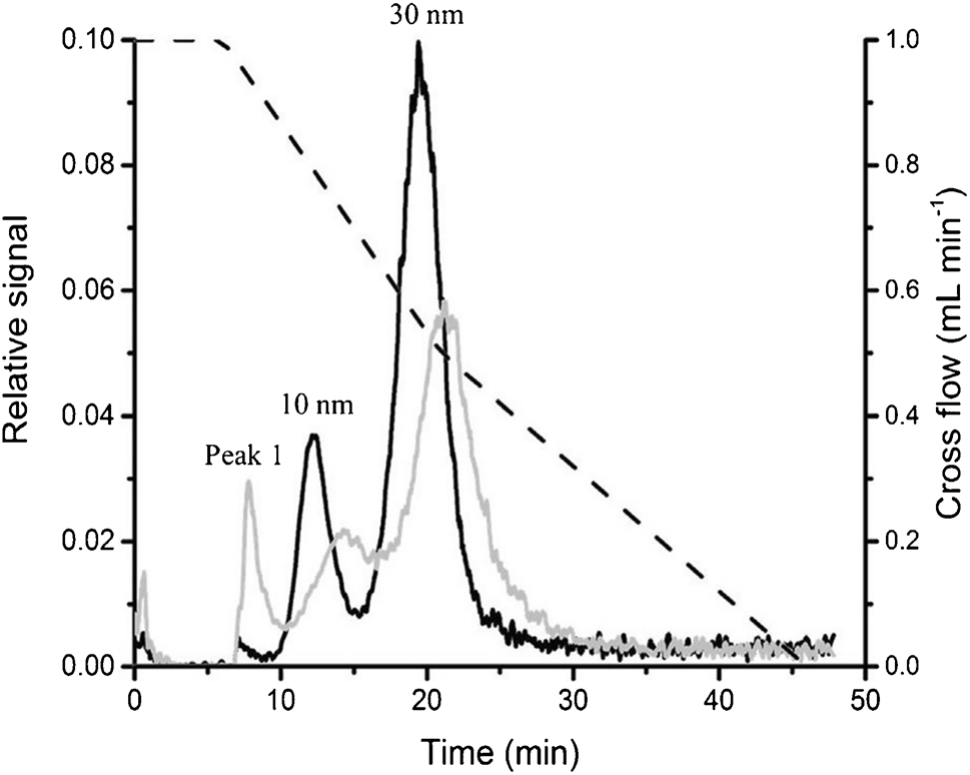
AF4-ICP-MS fractograms of an AuNPs mixture (10, and 30 nm) upon the exposure in DMEM-high glucose (supplemented with 10% FBS and antibiotics) (grey line), and without it (black line). The dash line represents the optimal cross flow program selected. (Reprinted with permission from López-Sanz et al. (2019), ^©^2019, Elsevier)

Non-chromatographic separation techniques, such as AF4 or CE, coupled to ICP-MS have also been used to follow the transformations in the NP hydrodynamic size. Thus, Bolea et al*.* [26] employed AF4-ICP-MS for the monitoring of protein corona formation. They focused on the detection and characterization of 15 nm AgNPs and dissolved Ag species in the cell culture medium DMEM-high glucose (supplemented with 10% FBS, and antibiotics) and HepG2 cells via AF4-UV-vis-ICP-MS. A shift towards to higher fractionation times was observed in presence of DMEM after 24 h of exposure time suggesting the protein corona effect. Related to HepG2, the higher Ag amounts found in the cells showed a low toxicity in comparison with Ag(I) as AgNO_3_ (16 to 1). A similar behaviour was also reported by López-Sanz et al*.* [80] for AuNPs of different sizes (10, 30, and 40 nm) in DMEM-high glucose by using the AF4-ICP-MS coupling and other complementary techniques. Under the optimal conditions, a mixture of 10 and 30 nm AuNPs without and with DMEM-high glucose could be separated in 30 min of analysis and AuNP transformations can be followed (Fig. 6). A clear displacement to higher fractionation times and a decrease in relative signal intensity were also observed for both AuNPs sizes in the presence of cell culture medium. With respect to the application of AF4-ICP-MS in in vivo toxicological tests, Coleman et al*.* [57] applied AF4-ICP-MS besides SP-ICP-MS for the study of AgNPs. The results of this work displayed a large peak in the fractogram which indicates an Ag fraction smaller than the nominal AgNP sizes in *L. variegatus* (in accordance with SP-ICP-MS data). The potential of CE-ICP-MS to track NP transformations within biological media with special attention to protein corona formation has also been proved in some in vitro experiments. Thus, Kruszewska et al*.* [44] developed a multiplatform including CE-ICP-MS for the monitoring of the transformations of various sized AuNP in MCF-7, and MCF-10a cells. AuNP-serum protein conjugates were disintegrated below 45 min of analysis. In addition, this research group also applied CE-ICP-MS/MS for the determination of superparamagnetic iron oxide NPs (SPIONPs) under simulated serum conditions [82]. The fact that no detectable peaks were found after 30 min of interaction suggested that most NPs and albumin molecules must be forming a conjugate.

When NPs interact with a biological media, they are prone to clustering via aggregation or agglomeration depending on the strength and reversibility of the process. Light-scattering techniques, such as DLS, are usually employed for the study of aggregation/agglomeration, although ICP-MS-based approaches can also be used to investigate NP aggregation. Thus, Hsiao et al*.* [20] assessed the transformations of AgNPs, and TiO_2_NPs in Neuro-2a cells by SP-ICP-MS confirming the formation of aggregates within these non-cancerous cells. In addition, the analysis of the cell lysates revealed a concentration and size dependency for both types of NPs. An aggregation process was also reported by Hawkins et al*.* [59] who conducted the identification and characterization of 20 nm AgNPs coated in citrate or PVP in the gastrointestinal tract, and gills of *Pimephales promelas* (fathead minnows) by AF4-ICP-MS. Regarding to the gastrointestinal tract, the analysis revealed an aggregation process ranging 40–70 nm and 40–55 nm for AgNPs coated in PVP and citrate, respectively. Respect to the gills, AgNPs did not agglomerate due to fewer biological interactions revealing a hydrodynamic size of 27 nm and 30 nm exposed to PVP and citrate, respectively.

Apart from the modifications in size potentially induced upon the NP dispersion in biological media, the assessment of other processes such as oxidation or ion release from nanostructures is of high interest to achieve an appropriate interpretation and management of the interactions between NPs and biological systems in future in vitro and in vivo toxicological studies. The information about these processes can be achieved using classical ICP-MS if it is combined with an adequate strategy for the separation of NP and ionic forms. In this sense, off-line separation techniques (i.e. ultrafiltration, ultracentrifugation, and dialysis) stand out for being fast and low-cost alternatives, which provide a fractionation by particle sizes and allow working with large volumes of sample. However, they can lead to erroneous results due to the electrostatic interaction and deposition of NPs in the membrane causing clogging or the formation of unwanted aggregates [92]. Thus, the use of online separation techniques hyphenated with ICP-MS becomes a better option for the study of NP ion release than previously mentioned off-line approaches. In this sense, using AF4-ICP-MS, Bolea et al*.* [26] reported a dissolved Ag species release from the nano-sized particles since they found an additional peak in the fractograms corresponding to AgNPs incubated in DMEM-high glucose culture medium. This finding, which is likely related to an oxidation process, has already been reported for AuNPs incubated in the same biological medium in studies also performed by AF4-ICP-MS [80] or others using HPLC-ICP-MS [39]. The same process was also observed by HPLC-ICP-TQ-MS for PtNPs after incubation in different cell culture media [84]. Apart from the evidence of oxidation processes induced by biological media, Álvarez-Fernández García et al*.* [68], which studied the degradation of 40 nm AuNPs in rat liver after intraperitoneal injection through several techniques including HPLC-ICP-MS, observed some dissolution processes. Thus, the results of this study confirmed not only the presence of 40 nm AuNPs but also with smaller size (6 ± 2 nm). Both AuNPs sizes were also found by TEM analysis, which suggested a degradation of these nano-sized particles accompanied by the detection of low-molecular Au species.

## Future perspectives and incoming developments

As previously presented in the “Quantitative information: NP cellular uptake” section, the quantification of NP cellular uptake has been mainly conducted by conventional elemental determination due to the numerous advantages of ICP-MS (i.e. multi-elemental and multi-isotopic capability, wide dynamic range, selectivity, accuracy, low detection limits in the ppt range) for elemental quantification of NPs. However, the analysis of some elements (i.e. Ti or Fe), and in extension their oxide NPs, is specially challenging in complex matrices due to different spectral and/or isobaric interferences affecting their most abundant isotopes. The alternatives initially proposed to overcome these limitations were based on the use of sector field (SF-ICP-MS) or single quad (ICP-Q-MS) instruments equipped with collision/reaction cells. The implementation of tandem ICP-MS (ICP-MS/MS) or ICP-TQ-MS systems has offered new possibilities for the control and removal of spectral interferences by introducing a reaction gas (oxygen or ammonia) which interacts with interfering ions. It has enabled to analyse the most abundant isotopes without interferences improving the sensitivity of analysis, but the potential of ICP-TQ-MS in nanotoxicological applications is still underexploited.

New modalities such as SP-ICP-MS have also become an interesting alternative for the study of NP characteristics because this tool allow to obtain simultaneous counting (number and mass concentration) and sizing (core size and number size distribution) information in a short time of analysis at trace level concentrations. However, its ability for NP characterization in biological systems is sometimes restricted by the complexity of the matrix which can be overcome using adequate strategies for NP separation, clean-up, purification, and preconcentration [92]. The performance of SP-ICP-MS is also limited by the lack of harmonization and standardization for the data acquisition and processing conditions. In this context, the estimation of transport efficiency becomes a crucial step, which usually involves the use of an adequate NP suspension certified, although other approaches based on the increase of the amount of sample entering the plasma or the measurement of the waste solution have also been explored [93]. It is also worth noting that a novel SP-ICP-MS strategy where the NP size determination is independent on the transport efficiency has been recently developed [94]. In this work, relative approximations based on the adequate fitting and normalization of SP-ICP-MS histograms were used to determine AuNP sizes after incubation with different concentrations of proteins, which demonstrates the applicability of the proposed method to control the NP modifications induced in complex samples. Difficulties in the application of SP-ICP-MS for the characterization of small NPs specially emerge when using ICP-MS of old generation due to the limitation in the data acquisition range (milliseconds). Currently, new detection systems can decrease the range to microseconds (μs). This choice allows to work with higher particle number concentration which maintains the low occurrence of 2-particle events and enables to obtain lower detection limits [95]. It is also remarkable the rapid transient method developed by Duffin et al*.* [96] for an efficient and highly sensitive detection of NPs based on the use of the ion arrival times as a means for particle detection and discrimination from background without the use of the typical predetermined integration windows. Moreover, Labied et al*.* presented an interesting proposal for the size measurement of ultrasmall NP (< 5 nm in the case of gadolinium NPs) [97]. This approach is based on the coupling of ICP-MS with Taylor dispersion analysis (TDA), which is an absolute, fast, and reliable technique for the measurement of hydrodynamic size using the previous determination of the molecule diffusion coefficients. The hyphenation of TDA to ICP-MS has been applied as a first insight in the toxicological field for the characterization of ultrasmall gadolinium containing NP in different biological fluids [97, 98] with a significant reduction in the LOD_size_ (reaching values even lower than 5 nm), but the potential of this novel approach should be further explored.

Although important advances have been achieved in SC analysis, especially since the development of emerging methods as LA-ICP-MS or SC-ICP-MS, some obstacles related to the lack of NP suspension certified or calibration standards, and limitations in the analytical throughput for applications in complex samples must be still overcome. Sample introduction is a key step for SC-ICP-MS since the introduction system must allow to reach high transport efficiency and preserve the cell integrity [77]. Thus, latter developments in SC-ICP-MS have been focused on the improvement of the introduction of cell suspensions into the ionization source with maximum transport efficiencies which can be achieved using new nebulizer or spray chamber systems [18, 77]. The use of alternative sample introduction systems like microdroplet generation reduces the size of droplets that can be efficiently introduced into ICP-MS configurations. Thus, a new interface including a pneumatic nebulizer and a microdroplet generator has been developed to determine the metal mass fraction and AuNP, AgNP, and CeO_2_NP number concentration in just 20 min of analysis [99]. The employment of this technology offers new opportunities in SC analysis [100]. Regarding LA-ICP-MS, this technique can be combined with other approaches to overcome its limitations and obtain complementary information. In this sense, new developments involve the combination of SP and LA-ICP-MS to achieve a size-selective mapping [100]. This approach has been already explored to image the size distributions of AuNPs in different mouse [101] or rat [102] tissues, the distributions of particulate and ionic released Ag in mouse organs [103], and also for the simultaneous determination of AgNPs and ionic forms in roots of sunflower [104], showing the uptake and transformation of ionic Ag to AgNPs Therefore, LA-SP-ICP-MS emerges as a method with high spatial resolution, sensitivity, and visualization capabilities and can become an interesting new tool for the investigation of NP translocation in biological processes improving the knowledge about the NP behaviour in in vivo conditions. Another relevant improvement in this field is based on the replacement of the typical quadrupole detector with a multiple mass analyser such as TOF (ICP-TOF-MS) or SF. These new ICP-MS-based systems could improve spatial resolution, sensitivity, and speed of data acquisition which would allow to perform a better registration of signals from single cells [105]. The capability for simultaneous analysis of this type of analysers offers attractive benefits specially for multicomponent analysis [8]. Thus, ICP-TOF-MS becomes an ideal detector for the simultaneous analysis of different NPs in mixtures of composite particles. The multi-elemental capacity of TOF analyser has also started to be considered in combination with SP analysis [106, 107], becoming an alternative specially to improve the discrimination between single particles and aggregates or engineered and natural NPs, which was still a limitation of traditional SP-ICP-MS analysis. Thus, the featured multiple-element capacity of SP-ICP-TOF-MS instruments is being gradually applied for the study of NPs in complex samples. Up to now, a matrix-independent quantification of NPs in terms of mass and number-based concentrations has already been reported [108]. Moreover, this platform has been applied for the first time to characterize NPs which contained Gd and Yb, improving the sensitivity (increased up to factor 27) and size detection limits (decreased by a factor of 3) [109]. The combination of ICP-TOF-MS with SC has also been explored for the study of different metal nanocluster (IrNC, AuNC, and PtNC)-labelled antibodies as specific tags for protein determination (hepcidin, metallothionein-2, and ferroprotein) in human ARPE-19 cells [111]. This analytical methodology allows to obtain the individual protein mass determination in single cells, as well as the relative cell volume, and the target concentration inside the cell. Nevertheless, even though the SP/SC-ICP-TOF-MS allow to overcome the limitations of SP-ICP-Q-MS for the monitoring of multiple isotopic signals from a single NP related to the settling time, the precision achieved in the elemental/isotopic ratio measurements is a bit limited. This precision can be improved using systems such as multiple collector mass spectrometers (MC-ICP-MS), but this option has been scarcely explored for the analysis of NPs up to now. This is because the time resolution of this approach is not high enough for the quantification of the transient signals registered in the SP mode. However, the combination of MC-ICP-MS and high-time resolution amplifiers (HTR) would enable to measure the elemental and isotope ratios from the transient signals obtained from the NPs. This combination (HTR-MC-ICP-MS) has been recently proposed and applied for the isotopic analysis of Pt from PtNPs [110]. Nevertheless, the potential of mass analysers distinct to quadrupole in the nanotoxicological applications is still an underexplored area, and further applications specially in this field would be needed.

Apart from the advances in the instrumental performance, the study of some metrological aspects (i.e. the demonstration of traceability, estimation of accuracy, and calculation of uncertainties) is still needed for the achievement of a thorough analytical NP characterization in samples of toxicological interest. One of the most important challenges is related to the NP quantification, especially due to the lack of NP suspension certified and adequate standards when using the classical approaches such as external calibration. In this sense, the quantification of NPs in complex samples could reach a higher metrological quality by incorporating tools such as the isotopic dilution analysis (IDA). This approach can be used not only in conventional ICP-MS analysis but also in combination with other modalities such as SP-ICP-MS or AF4-ICP-MS. Only few investigations have been conducted so far using IDA for the characterization of NPs (mainly AgNPs) [111–113], but its application to complex biological matrices has been scarcely explored yet. One of the main limitations of IDA-SP-ICP-MS approaches is the long measurement cycles needed for the acquisition of both isotopes. For these reason, recent investigations have evaluated the performance of SP-ICP-TOF-MS in combination with IDA for the size determination of AgNPs [114] or PtNPs [115]. In both cases, the use of a TOF mass analyser has enabled the simultaneous detection of the isotopes leading to a more precise determination of isotope ratios. Concerning the application to toxicological assays, Zheng et al*.* [116] has proposed an interesting new approach based on IDA, called single-cell IDA (SCIDA). As a case of study, macrophage cells were chosen as a model to study the uptake of AgNPs (20 nm) by SCIDA LA-ICP-MS. The results showed an average uptake of 396 fg Ag cell^−1^ from 1100 single cells, which is consistent with the data obtained by ICP-MS analysis (393 fg cell^−1^). Moreover, no effects of the cell matrix in the isotopic ratio measurements were observed. Hence, this work demonstrates the potential of SCIDA for the determination of NPs at single-cell level since both the analytical throughput and the accuracy in NP quantification have been improved. Nevertheless, this promising tool must be further explored before it can be routinely applied for the study of NP-cell interactions in toxicological assays.

In order to achieve a comprehensive understanding of NP internalization, the distinction between the material internalized from the material bound to the cell surface is a critical point that must be carefully addressed. Different methods including acid-washing or the use of proteases as trypsin can serve to dissociate surface-bound material from the cell membrane. This latter is the most adequate method to remove NPs bound to receptors if they are attached to proteins since the enzyme can digest cell surface proteins. Nevertheless, to address if the NPs have been internalized or only adsorbed into the cell surface not only specific sample treatments but also an adequate combination of ICP approaches with imaging techniques (i.e. microscopy or imaging flow cytometry) would be required to thoroughly assess the NP internalization [76]. With regard to the investigation of the transformations after the cellular uptake or the contact of NP with a biological media, the identification, and study of proteins, as well as their abundance, and affinity are essential (e.g. unbound proteins present in the biological media must be removed before the protein corona study). Specific methods such as purification, isolation (i.e. centrifugation, SEC), and magnetic separation are usually preferred [117]. Furthermore, the key role developed by the instrumental separation techniques coupled to ICP-MS is undoubtless. On one hand, approaches based on separation by HPLC, or CE becomes an attractive option due to their simplicity and cost-effectiveness. On the other hand, the importance of AF4 for the fractionation of NPs has already been addressed. New trends and developments of AF4 systems are related to the combination of this separation technique with an electrical module enabling the application of an electrical field in addition to the regular cross-flow. Thus, the modality of electrical AF4 (EAF4) becomes a promising tool for the simultaneous study of NP size and electrical properties. This novel separation approach has already been tested in combination with multiple detectors such as UV-vis and MALS [118–120], but the potential of the combination with ICP-MS has been scarcely explored yet. As far as we known, only Fernandez-Trujillo et al*.* [121] have recently proposed a EAF4 multi-detector array platform (including detection by ICP-TQ-MS) for the characterization of different NPs. However, this technique is in its infancy and a robust implementation is still required, so further works about the potential of EAF4-ICP-MS for the nano characterization in toxicological assays would be therefore interesting and needed.

To conclude, it is worth to point out that the exhaustive characterization of NPs in complex toxicological matrices requires distinct and complementary information, which can be obtained by using the different approaches described throughout this review. It demonstrates not only the featured contribution of the ICP-MS-based systems in toxicological studies, but also that a complete set of analytical techniques is needed for the full understanding of NP toxicity and the NP-biomolecules or cell interactions.
